# The value of repeated CT in monitoring the disease progression in moderate COVID-19 pneumonia: A single-center, retrospective study

**DOI:** 10.1097/MD.0000000000025005

**Published:** 2021-03-12

**Authors:** Yang Gao, Yuxiong Hu, Junteng Zhu, Huan Liu, Rongxian Qiu, Qunying Lin, Xiongzhi He, Hai-Bin Lin, Shiming Cheng, Guangxi Li

**Affiliations:** a Division of Pulmonary Medicine, Guang’anmen Hospital China Academy of Chinese Medical Sciences, Beijing; b Department of Pulmonary and Critical Care Medicine; c Department of Rehabilitation Medicine, The Affiliated Hospital of Putian University, Putian, Fujian; d Division of Radiology, Guang’anmen Hospital, China Academy of Chinese Medical Sciences, Beijing; e Department of Infectious Diseases and Hepatology; f Department of Osteology, The Affiliated Hospital of Putian University, Putian, Fujian; g Chinese Antituberculosis Association, China; h Division of Pulmonary and Critical Care Medicine, Mayo Clinic, Rochester, MN.

**Keywords:** COVID-19 pneumonia, CT scan, follow up, treatment outcome

## Abstract

The role of thoracic CT (computerized tomography) in monitoring disease course of COVID-19 is controversial. The purpose of this study is to investigate the risk factors and predictive value of deterioration on repeatedly performed CT scan during hospitalization.

All COVID-19 patients treated in our isolation ward, from January 22, 2020 to February 7, 2020, were reviewed. Patients included were categorized into RD (Radiological Deterioration) group or NRD (No Radiological Deterioration) group according to the manifestation on the CT routinely performed during the hospitalization. All clinical data and CT images were analyzed.

Forty three patients were included in our study. All are moderate cases with at least 4 CT scans each. Eighteen (42.9%) patients had radiological deteriorations which were all identified in CT2 (the first CT after admission). Patients in RD group had lower leukocyte count (*P* = .003), lymphocyte count (*P* = .030), and higher prevalence (*P* = .012) of elevated C-reactive protein (CRP) at admission. NRD patients had a lower prevalence of reticulations (*P* = .034) on baseline CT (CT1, performed within 2 days before admission) and a longer duration between symptom onset and the time of CT2 (*P* < .01). There was no significant difference in hospital stay or fibrotic change on CT4 (follow-up CT scan performed 4 weeks after discharge) between 2 groups. Shorter duration between symptom onset and CT2 time (odds ratio [OR], 0.436; 95% confidence interval: 0.233–0.816; *P* < .01) and lower leukocyte count in baseline evaluation (OR, 0.316; 95% CI: 0.116–0.859; *P* < .05) were associated with increased odds of radiological deterioration on CT image during hospitalization.

For moderate COVID-19 patients, the value of routinely performed CT during the treatment is limited. We recommend avoiding using CT as a routine monitor in moderate COVID-19 patients.

## 1 Introduction

First reported in Wuhan, China, the COVID-19 pandemic is one of the most devastating known epidemics in recent centuries, affecting 10,533,779 people and claiming 512,8421 global deaths, as of July 3, 2020.^[1]^ With no definitive treatment or available vaccine in sight, these numbers are still increasing exponentially, creating havoc for the health and financial systems of the world.

Radiological evaluations, particularly thin slice chest computed tomography (CT) scan, plays an essential role in diagnosis, management, and follow-up of COVID-19 case, recommendations on the use of CT for the screening and monitoring of COVID-19, however, were controversial.^[2–4]^ Since the outbreak, we have treated 52 COVID-19 patients in the isolation wards of the Affiliated Hospital of Putian University. After uniformly formulated treatment algorithm based on the guidelines of 2019-nCoV (Trial Version 5) proposed by the China National Health Commission,^[3]^ all patients had been cured and discharged, however, nearly one-third of them exhibited radiological deterioration on the routine CT evaluation about 10 days after admission, without obvious symptomatic aggravation. Does the radiological deterioration authentically indicate disease progression, is it because imaging lags behind clinical manifestation, or is it just the natural imaging evolution of COVID-19 pneumonia? It exerts substantial stress and dilemma on our clinical decision, especially in the circumstance of dealing with an unknown, fiercely infectious disease. Should we modulate the treatment? To what extent can the routine CT evaluation provide valuable information to facilitate clinical decisions?

To answer the questions above and investigate this phenomenon, we retrospectively reviewed all COVID patients treated in our hospital, all of them have serial CT evaluations during the whole disease course as well as follow up period. A longitudinal study was conducted to analyze the evolution of CT findings in disease progression and recovery.

## 2 Methods

### 2.1 Study design and participants

In this retrospective study, all patients with laboratory-confirmed COVID-19 who were admitted to the our isolation ward of the Affiliated Hospital of Putian University during the pandemic (from the first admission on January 22, 2020 to February 7, 2020, when the last patient was discharged home and the isolation ward was closed) were reviewed. Diagnostic criteria for COVID-19 were based on the diagnosis and treatment protocols from the National Health Commission of the People's Republic of China.^[3]^ Severe acute respiratory syndrome coronavirus 2 infection was confirmed by a positive result on real-time reverse-transcriptase–polymerase-chain-reaction (RT-PCR) assay of nasal and pharyngeal swab specimens. Patients with missing clinical or CT records on admission were excluded.

To maximize the efficiency of the limited medical resources, all COVID-19 cases at different clinical grade are assigned to specific hospitals or wards. Our isolation ward treated moderate cases only. The classification criteria of clinical grading were as follows^[3]^: mild, subtle or mild clinical symptoms without pneumonic CT finding; moderate, fever or respiratory symptoms and pneumonia on CT images; severe, with any of the followings: respiratory distress with respiratory rate >30/second, resting-state oxygen saturation <93%, or oxygenation index (calculated by the partial pressure of oxygen/fraction of inspired oxygen <300 mm Hg (1 mm Hg = 0.133 kPa); critical, with any of the followings: respiratory failure and mechanical ventilation needed, shock, or combination with other organ failure needing intensive care unit.

The protocol was approved by the Ethics Committee of the Affiliated Hospital of Putian University. Because of the urgent need to collect data on this emerging infectious disease, the requirement for written informed consent was waived.

### 2.2 Clinical Data and CT image acquisition

All the clinical data on epidemiology, signs and symptoms, laboratory test results, hospital stay were extracted from electronic medical records. Chest CT (HRCT) was performed for all patients.

Each patient included had at least a series of 4 CT evaluations: CT1: Baseline CT performed within 2 days before admission; CT2: the routinely performed CT scan on approximately the 10th day during hospitalization; CT3: CT scan performed 1–2 days before discharge (if the hospital stay was longer than 3 weeks); CT4: 4 weeks after discharge for follow-up. The CT examinations were performed according to standard non-contrast chest CT protocols. All patients were scanned in the supine position with breath holding at the end of inhaling. The chest CT scanner models and scanning parameters were as following: GE CTLightSpeed, SOMATOM Emotion 16, SOMATOM Definition Flash, tube voltage 130 kV, tube current 25 mA, pitch 1.0, rotation time ranging from 0.5 seconds to 0.75 seconds, slice thickness 5 mm, with 1 mm or 1.5 mm section thickness for axial, coronal, and sagittal reconstructions.

Symptoms reported on daily ward round were extracted from the electronic medical records, along with C reactive protein (CRP) were used as indicators of disease status and treatment response.^[5]^ Pulmonary function tests were not accessible during the pandemic, therefore we used CT4 as a follow-up assessment by identifying the presence of fibrotic change evidence which is defined as parenchymal bands, irregular interfaces (bronchovascular, pleural, or mediastinal), thickened interstitium, and traction bronchiectasis.^[6]^

### 2.3 CT image analysis and quantification

CT images were independently reviewed by 2 experienced radiologists (S. Shi with 10-year experience and H. Liu with 11-year experience). The consensus was applied as the final decision. For disagreement between the 2 primary radiologist interpretations, a third radiologist (XD Yang) with 20-year experience adjudicated a final decision. To minimize the bias, all 3 radiologists were blinded to the clinical data of the patients.

The predominant lung parenchymal lesions seen on CT images were classified into the following 10 patterns^[7,8]^: ground-glass opacities (GGO; increased attenuation without obscuration of the underlying lung vessels), consolidation (homogeneous increased intensity of lung parenchyma with obscuration of the underlying vessels), centrilobular nodules, traction bronchiectasis (irregular or distorted dilated airways seen in areas of fibrosis), mediastinal lymphadenopathy, reticulation (interlobular or intralobular irregular septal thickening), tree-in-bud sign, microvascular dilation sign (dilated small vessels in the lesion), fibrotic streaks (an irregular strip shadow), a subpleural line (an arc-shaped linear shadow 2 to 5 cm in length appearing parallel to the chest wall). Distribution of lesions was classified as peripheral, central and peripheral. Pleural thickening and pleural effusion were recorded as well. Abnormalities not related to COVID-19 pneumonia was ruled out.

The radiologists estimated the lesion volume in each lung segment as a percentage of the whole lung segment, and the percentages of the involved area in each segment were scored using the following 3-grade scale: 0, absent; 1, less than 50%; 2, more than 50%. Scores from the whole lung were summarized to get the total scores of each patient. A longitudinal comparison among serial CT evaluations of each patient was conducted. Any increase of consolidation or ground-glass opacity (GGO) in lesion volume, density, or distribution (number of lesions, new-developed lesions) were defined as radiological deterioration (RD).

### 2.4 Statistical analysis

Continuous variables compatible with normal distribution were presented as the mean (standard deviation [SD]) or median (interquartile range [IQR]) if incompatible to normal distribution. Differences between groups (presence and non-presence of radiological deterioration during treatment) were compared using Student *t* test or Mann–Whitney *U* test depending on distributions. Categorical variables were presented as n (%) and compared using χ^2^ or Fisher exact test. We used multivariable logistic regression models to identify CT and clinical risk factors for the radiological deterioration during treatment. We performed a sensitivity analysis of the completed cases. We set statistical significance at the 2-tailed *P* < .05. All analyses were performed using SPSS (version 22.0. IBM Corp. Armonk, NY).

## 3 Results

Fifty three laboratory-confirmed COVID-19 patients in total had been treated in the isolation ward of the Affiliated Hospital of Putian University. All were moderate COVID-19 pneumonia cases at admission and were cured eventually. Ten patients without baseline CT scans or clinical records were excluded. We included 43 patients in the final analysis. The demographic and clinical characteristics were summarized in Table 1. Among the included patients (mean age, 44.35 ± 13.44 years, range, 15–76 years), 26 were men (60.5%). All patients reported symptoms at admission, with fever being the most common symptom (34 patients, 79.1%) followed by cough noted in 30 patients (69.8%). Moreover, 17 (39.5%) patients had comorbidities at the disease onset with hypertension being the most common.

**Table 1:** Baseline demographic and clinical characteristics of the included patients.

Characteristics	All patients (n = 43)	RD Group (n = 18)	NRD group (n = 25)	*P* value
Age (y, mean ± standard deviation)	44.35 ± 13.44	46.89 ± 13.60	42.52 ± 13.29	.298
Male Gender (%)	26 (60.5%)	11 (61.1%)	15 (60.0%)	.941
BMI	23.39 ± 2.76	23.73 ± 3.20	23.13 ± 2.41	.495
Initial symptoms				
Fever^†^ (%)	34 (79.1%)	15 (83.3%)	19 (76.0%)	.560
Low fever (37.3–38°C)	15 (34.9%)	4 (22.2%)	11 (44.0%)	.139
Medium fever (38–39°C)	18 (41.9%)	10 (55.6%)	8 (23.0%)	.122
High fever (>39°C)	1 (2.3%)	1 (5.6%)	0 (0%)	.233
Body temperature	37.75 ± 0.75	37.88 ± 0.75	37.65 ± 0.74	.321
Cough (%)	30 (69.8%)	12 (66.7%)	18 (72.0%)	.707
Fatigue (%)	5 (11.6%)	4 (22.2%)	1 (4%)	.066
Dyspnea (%)	2 (4.7%)	2 (11.1%)	0 (0.0%)	.222
Sore throat (%)	3 (7.0%)	2 (11.1%)	1 (4%)	.367
Nasal congestion and runny nose (%)	7 (16.3%)	4 (22.2%)	3 (12.0%)	.382
Loss of appetite (%)	3 (7.0%)	2 (11.1%)	1 (4%)	.367
Laboratory test findings				
Leukocyte count (10^9^/L)	5.65 ± 2.03	4.61 ± 1.56	6.40 ± 2.02	.003^∗^
Lymphocyte count (10^9^/L)	1.67 ± 0.67	1.41 ± 0.63	1.86 ± 0.65	.030^∗^
Increased hs-CRP (%)	19 (44.2%)	12 (66.7%)	7 (28.0%)	.012^∗^
Comorbidities (%)				
Cardiovascular disease^‡^	6 (13.9%)	2 (11.1%)	4 (16.0%)	.684
Diabetes mellitus type II	4 (9.3%)	2 (11.1%)	2 (8.0%)	.729
Liver disease^§^	4 (9.3%)	2 (11.1%)	2 (8.0%)	.729
Hyperthyroiditis	1 (2.3%)	0 (0.0%)	1 (4.0%)	.391
Allergic rhinitis	1 (2.3%)	0 (0.0%)	1 (4.0%)	.391
Hospital stay (days)	21 (17)	24 (18.75)	18 (15.5)	.217
Time from symptom onset to hospital admission (days)	5 (7)	4 (5)	5 (6)	.258

∗ *P* < .05.

† Maximum body temperature since symptoms onset. Fever was defined as axillary temperature of at least 37.3°C.

‡ Cardiovascular disease included hypertension, coronary artery disease.

§ Liver disease included cirrhosis, hepatitis B.

**Figure 1:**
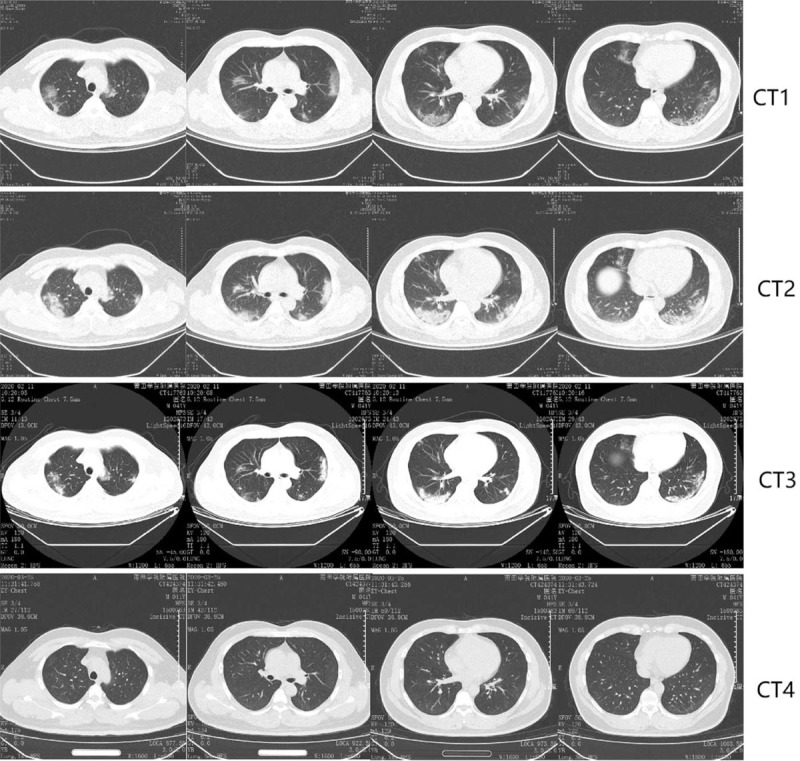
Representative chest computed tomography scan images in RD group. A 42-year-old female with confirmed 2019-nCoV infection, moderate type at baseline. Patient had no exposure history to Wuhan but a close contact history with confirmed cases. The onset symptom was fever (38.5°C). CT1, performed one day before admission, shows peripheral distributed slight slice-like shadow and ground-glass opacities with fuzzy edges in both lungs. During the treatment, the CT2 performed on the 6th day of hospitalization presented deterioration (enlarged lesions with increased density). The patient responded well to the treatment and was discharged on the 14th day after admission. Obvious absorption of lesions in both lungs was observed on CT3, which was performed 2 days before discharge. CT4 was the follow-up CT one month after discharge. CT1-4 scans were performed on February 1, 7, 11, 2020 and March 25, 2020, respectively.

**Figure 2:**
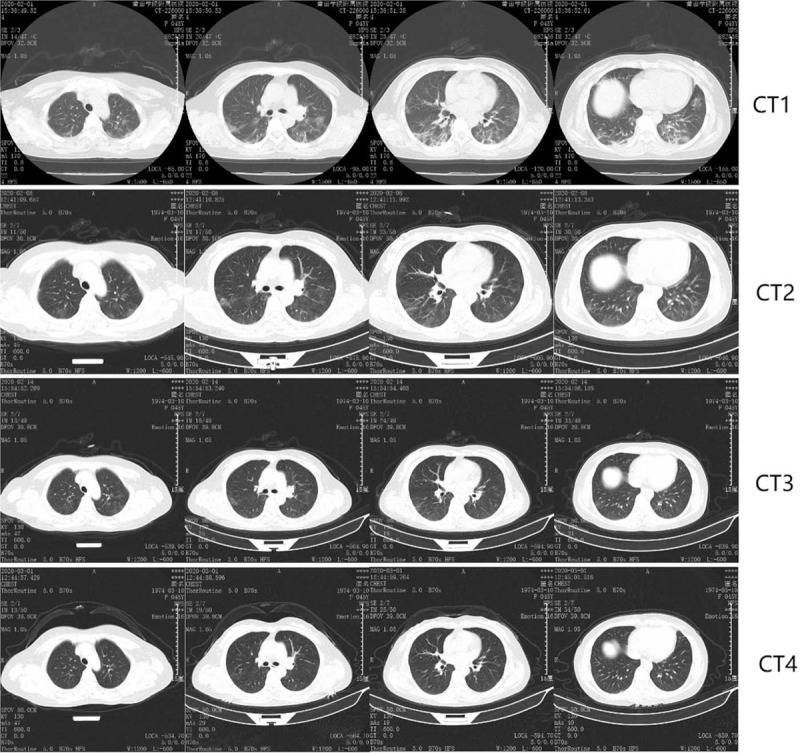
Representative chest computed tomography scan images in NRD group. A 46-year-old female with confirmed moderate 2019-nCoV pneumonia, presented with dry cough but a normal temperature (36.8°C) at admission. She had no exposure history to Wuhan but a close contact history with confirmed cases. The Baseline CT1 showed a multifocal, peripherally distributed GGOs and fibrotic streaks. On the 8th day of hospitalization, CT2 revealed shrinking lesions with increased transparency indicating alleviation. The patient response well to the treatment and was discharged home 7 days later. CT3 was performed 1 day before discharge, CT4 was the follow-up CT on the 16th day after discharge. CT1-3 scans were performed on February 1, 8, 14, 2020 and March 12, 2020, respectively.

During the hospitalization, 18/43 (42.9%) patients had radiological deterioration (Fig. 1, categorized as RD group), all were identified in the first CT evaluation (CT2) which was routinely performed approximately 10 days after admission. As shown in Table 1, compared with patients who did not experience radiological deterioration (Fig. 2, categorized as NRD group), patients in RD group had lower leukocyte count (4.61 ± 1.56 vs 6.40 ± 2.02, *P* = .003), lymphocyte count (1.41 ± 0.63 vs 1.86 ± 0.65, *P* = .030) and higher prevalence (12, 66.7% vs 7, 28.0%, *P* = .012) of elevated C-reactive protein (CRP) at admission. There was a trend that medium (38–39°C) to high (>39°C) fever [10 (55.6%) vs 8 (23.0%), 1 (5.6%) vs 0 (0%)] and fatigue symptom [4 (22.2%) vs 1 (4%)] were more commonly seen in RD patients at baseline, but the difference was not statistically significant (*P* = .122, *P* = .233, *P* = .066, respectively). All patients responded well to the treatment and were cured, there was no significant difference in hospital stay or fibrotic change identified in CT4 between 2 groups.

Baseline CT scans (CT1) were performed at admission or within 2 days before. All 43 patients had pneumonia findings with GGO, consolidation, microvascular dilation sign, fibrotic streaks being the most commonly seen patterns. Tree-in-bud signs and pleural involvements were not found in our cohort. GGO and peripheral distribution in all the patients was observed generally. CT quantitative evaluation of pulmonary lesions revealed that there is no significant difference between GGO and consolidation scores in patients with RD and those in patients without (*P* = .951, *P* = .433, respectively). Compared with patients with RD, those without RD tend to have a lower prevalence of reticulations (*P* = .034) on CT1. Fibrotic streaks and subpleural lines tend to be more common in the NRD group of patients but the difference was not significant (*P* = .068, *P* = .106 respectively). Interestingly, patients in the RD group had a shorter duration between symptom onset and the time of CT2 (*P* = .000), as shown in Table 2. Moreover, although patients exhibit RD on CT images, the follow-up CRP 1 week after admission, close to the time of CT2, decreased significantly compared with baseline value (Tables 3 and 4).

**Table 2:** Image Characteristic of baseline HRCT.

Image Characteristics	All patients (n = 52)	RD group (n = 18)	NRD group (n = 25)	*P* value
Transverse distribution				.385
Peripheral	33 (76.7%)	15 (83.3%)	18 (72.0%)	
Central and peripheral	10 (23.3%)	3 (16.7%)	7 (28.0%)	
Craniocaudal distribution				.653
Upper lung predominant	3 (7.0%)	1 (5.6%)	2 (8.0%)	
Lower lung predominant	22 (51.2%)	8 (44.4%)	14 (56.0%)	
No craniocaudal predominance	18 (41.9)	9 (50.0%)	9 (36.0%)	
Sagittal distribution				.090
Anterior lung predominant	0 (0.0%)	0 (0.0%)	0 (0.0%)	
Posterior lung predominant	32 (74.4%)	11 (61.1%)	21 (84.0%)	
No Sagittal predominance	11 (25.6%)	7 (38.9%)	4 (16.0%)	
Ground glass opacity (GGO)	42 (97.7%)	18 (100%)	24 (96%)	.391
GGO score	7 (10)	8 (12)	6 (11)	.951
Consolidation	20 (46.5%)	8 (44.4%)	12 (48.0%)	.818
Consolidation score	1 (2)	1 (1.25)	1 (3.5)	.433
Centrilobular nodules	0 (0%)	0 (0%)	0 (0%)	–
Traction bronchiectasis	2 (4.7%)	1 (5.6%)	1 (4.0%)	.811
Mediastinal lymphadenopathy	2 (4.7%)	0 (0.0%)	2 (8.0%)	.219
Pleural effusions	0 (0%)	0 (0%)	0 (0%)	–
Reticulation	3 (7.0%)	3 (16.7%)	0 (0.0%)	.034
Tree-in-bud sign	0 (0%)	0 (0%)	0 (0%)	–
Microvascular dilation sign	28 (65.1%)	13 (72.2%)	15 (60.0%)	.407
Fibrotic streaks	26 (60.5%)	8 (44.4%)	18 (72.0%)	.068
Subpleural line	7 (16.3%)	1 (5.6%)	6 (24%)	.106
Time from symptom onset to CT2^†^	14 (6)	12 (4)	17 (6)	.000^∗^
Fibrotic changes on follow-up CT	24 (55.8%)	11 (61.1%)	13 (52%)	.553

GGO score, Consolidation score, Days from symptom onset to CT2^†^ are presented as median (interquartile range: 25th percentile – 75th percentile), others are presented as n (%).

∗ *P* < .05.

† CT2 is the first routine CT evaluation during hospitalization. All radiological deterioration occurred in CT2. NRD = non-radiological deterioration, RD = radiological deterioration.

**Table 3:** Comparison of CRP value in RD group.

	RD (n = 18)		
CRP value categories (mg/L)	CRP (mg/L) baseline (on admission)	CRP (mg/L) Closest prior to CT2	*P* value
<10	7	13	*P* = .042
10–30	7	4	
30–50	2	0	
50–100	2	1	
> 100	0	0	

**Table 4:** B Comparison of CRP value in NRD group.

	NRD (n = 25)		
CRP value categories (mg/L)	CRP (mg/L) baseline (on admission)	CRP (mg/L) Closest prior to CT2	*P* value
<10	21	23	*P* = .371
10–30	3	2	
30–50	1	0	
50–100	0	0	
>100	0	0	

There was almost no evident symptomatic aggravation that was recorded at the time when CT2 was performed, except only 1 patient in the RD group reported worse dyspnea. The values of CRP test closest before CT2 were obtained and compared with the baseline counterpart to assess the change of disease status and treatment response. We converted CRP values into categorical variables using the interval of <10 mg/L, 10–30 mg/L, 30–50 mg/L, 50–100 mg/L, >100 mg/L, because the value did not reveal as an exact number while it was less than 10 mg/L. A significant decline was observed on CRP of the RD group (*P* = .042, Table 3) while the values of NRD patients were stable (*P* = .371, Table 4)

**Figure 3:**
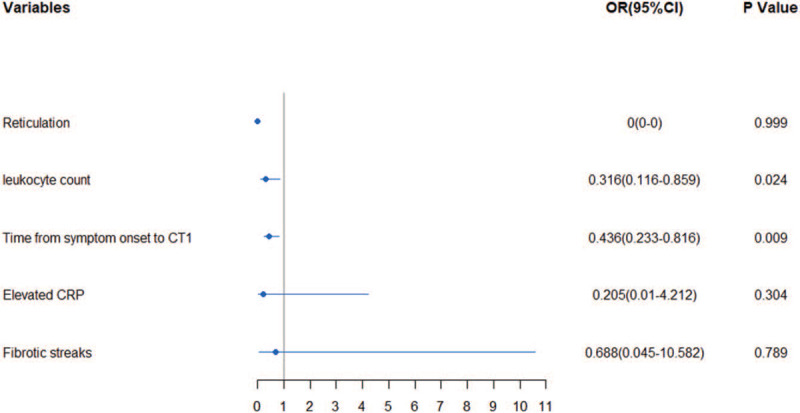
Multivariable logistic regression to identify factors associated with adiological deterioration on CT image during hospitalization. Shorter duration from symptom onset to CT2 and lower leukocyte count of baseline were more likely to have radiological deterioration on CT image.

Multivariable logistic regression was performed based on the univariate analysis above. Five statistically significant variables: leukocyte count, the prevalence of elevated CRP, reticulations on baseline CT, the time from symptom onset to CT2, and fibrotic streaks were included in the model. As illustrated in Figure 3, shorter duration between symptom onset and CT2 (odds ratio [OR], 0.436; 95% confidence interval [CI: 0.233–0.816; *P* = .009) and lower leukocyte count in baseline evaluation (OR, 0.316; 95% CI: 0.116–0.859; *P* = .024) were associated with increased odds of radiological deterioration on CT image during hospitalization.

## 4 Discussion

Consistent with prior studies, in our cohort, bilateral, peripheral, and posterior distributed GGOs with or without consolidations are the most prevalent chest CT findings. Reticulations are more commonly found in baseline CT of patients with radiological deterioration. GGO, due to partial filling of alveoli airspaces or interstitial thickening, was believed to be the predominant CT abnormality in COVID-19 patients at an early stage and preclinical patients.^[9]^ It usually becomes more diffused and increases in density within 1 to 3 weeks to become consolidated, indicating an accumulation of inflammatory cellular exudate in the alveoli and adjoining ducts and generate a consolidation pattern which has been reported as the second predominant feature in COVID-19 patients within the first few days of disease onset.^[10]^ Reticulation was reported to be more common in COVID-19 cases compared with other viral pneumonia (56% vs 22%, *P* < .001),^[9]^ in our cohort, however, probably because of the different standard, the prevalence was only 7% (3 patients). Although being cured, all 3 patients with reticulations were found in the RD group, radiologically in accordance with a previous study reporting reticulation indicated an advanced disease stage.^[11]^ Similar as prior study,^[12]^ leukocytopenia, lymphocytopenia, and elevated CRP were common in our patients. It is hypothesized that 2019-nCoV predominantly involves lymphocytes, especially T lymphocytes, Virus damage respiratory mucosa and make their way to infect other organs, induce a series of immune responses and cytokine storm, involve peripheral white blood cells including lymphocytes.^[13]^

By conducting this retrospective study, we sought to analyze how the radiological deterioration would impact the outcome or prognosis and identify the imaging and clinical risk factors associated with radiological deterioration during hospitalization in patients with moderate COVID-19 pneumonia. After univariable analysis and multivariable logistic regression, we identified a shorter duration between symptom onset and the time of CT2, lower leukocyte count at admission were associated with higher odds of radiological deterioration during treatment. Let us get back to the questions raised in the beginning.

### 4.1 Does the radiological deterioration authentically indicate disease progression?

For moderate COVID-19 pneumonia patients, probably not. During the hospitalization, CT2 scans were performed routinely for all patients 10 days after admission rather than clinical status originated, almost no symptomatic aggravation was reported at the time of CT2. Moreover, in our cohort, although patients with radiological deterioration had significantly lower leukocyte count, lymphocyte count and higher prevalence of CRP, which suggest this group of patients might have relatively worse disease status at admission, after a uniformly formulated and guidelines based treatment^[3]^ consist of recombinant human interferon α2b (aerosol inhalation), lopinavir and ritonavir tablets (200 mg/50 mg twice daily, orally), Qingfeipaidu decoction (Chinese Medicine, 200 ml twice daily, orally), Corticosteroid treatment and antibiotic treatment when appropriate, all patients had a good response to the treatment regimen and were cured eventually. There was no significant difference in hospital stay or fibrotic change in a 4 week-follow-up CT scan between patients with radiological deterioration and those without.

Since included patients are all in moderate stage at baseline and none had progressed to the severe or critical stage, our laboratory tests were limited within simple and less invasive modalities. Artery blood gas, immunological parameters, and cytokines did not apply to most patients, and the change of leukocyte count would also be potentially interfered with secondary bacterial infection and corticosteroids. Therefore, we used CRP as an indicator to dynamically reflect disease status and treatment response.^[5]^ Contributing to activate the complement and enhance phagocytosis, thus clearing the pathogenic microorganisms invading the body, CRP levels can reflect the level of inflammation and relatively independent from confounding factors such as age, sex, and physical condition, can be used as an important index for the diagnosis and assessment of pulmonary infectious diseases.^[14–16]^ As we can see from table 3, CRP levels of all patients including patients with radiological deterioration maintained stable or decreased at the time of CT2, the deterioration shown on CT images are at odds with disease status.

### 4.2 How to explain this phenomenon?

We hypothesis that it might because of the hysteresis of imaging findings, the absorption of lung infiltration usually lags behind the improvement of clinical symptoms. This phenomenon was also documented in pneumonia caused by severe acute respiratory syndrome and H1N1.^[17,18]^ It is commonly seen in almost all sorts of diseases that contribute to lung infiltration during clinical practice, although rarely described literally.

Another possible explanation might be the natural evolution of COVID-19. This possibility was based on a presumption that CRP and stable symptom reported could not authentically reflect the disease status. Admittedly, although numerous laboratory studies and clinical trials are full speed ahead, there is still no definitive treatment for COVID-19. Besides, like other viral infected diseases, COVID-19 could be self-limited or self-healing in a certain proportion of patients.^[19]^ Prior studies states CT image of COVID would reach a peak at 6 to 11 days after symptom onset.^[20,21]^ It seems shorter than the time course in our cohort.

### 4.3 To what extent can the routine CT evaluation provide valuable information to facilitate clinical decisions?

Although recently published studies argued that CT has high sensitivity and specificity in the evaluation of suspected COVID-19 pneumonia,^[9,22,23]^ the role of CT in COVID-19 management is still of much debate. Theoretically, there are indeed different trends in CT patterns of infections caused by divergent pathogens.^[24]^ In clinical practice, however, to generate a diagnosis merely or mainly from images is not practical. The imaging characteristics of COVID-19 pneumonia, bilaterally and peripherally distributed ground-glass opacities that are predominantly located in the lower lobes with or without consolidation,^[8,25,26]^ are not unique enough as a definitive criterion in the differential diagnosis. Instead, they may present in any acute lung injury associated with numerous infectious and noninfectious inflammatory conditions.^[27]^ Instead of being a predominant screening or diagnostic modality rivaling reverse transcription-polymerase chain reaction (RT-PCR), it serves as an adjunct but irreplaceable tool integrated with other tests and epidemiological history in COVID-19 diagnosis.

Inconsistent with a prior study conducted by Wei Zhao, indicating follow-up CT changes during the treatment can help evaluate the treatment response of patients with COVID-19 pneumonia,^[28]^ our study suggests in moderated COVID pneumonia patients, deterioration in routinely performed CT scan during treatment neither authentically reflect disease status nor predict treatment outcome or prognosis. We chose to stick to clinical manifestations which we believe to be more authentically, real-time reflect disease progression. We did not modulate our treatment measures for almost all patients with radiological deterioration, except 1 patient reported aggravated dyspnea was given corticosteroids plus prior treatment, all patients were cured eventually. From this perspective, patients with moderate COVID-19 in our cohort seem unlikely to benefit from routine CT evaluation during treatment. Moreover, immune cells especially lymphocytes are vulnerable to radiation damage^[29,30]^ and the pathogenesis of severe acute respiratory syndrome coronavirus 2 also involves the immune system.^[31]^ Therefore, the patient's immune system would probably take a double hit while repeatedly performing CT scan.

In a word, based on the present study, as for patients with moderate COVID-19 pneumonia, we are inclined to believe that the information generated from routinely performed CT scans during treatment is limited and of poor risk-reward ratio.

Admittedly, our study has several limitations. First, this was a single-center retrospective study with only 43 patients with moderate disease included. Some more severe cases might be transferred to other hospitals that generate a selection bias. Second, some important laboratory test results and parameters, such as arterial blood gas, virus load (cycle threshold value), follow-up pulmonary function tests were not analyzed in our study due to the limited data availability. Studies of larger sample size and comprehensive data acquisition are needed to better assess the value of the CT scan in COVID-19 disease monitoring

## 5 Conclusion

In conclusion, for moderate COVID patients, the routinely performed CT scan during the treatment could neither help evaluate the treatment response nor predict progress. We recommend avoiding using CT as a routine monitor in treating moderate COVID patients. Especially for those without any indications for disease progression. Multicenter, large-scale research need to verify the results in the future.

## Acknowledgments

We thank Dr. Shan Shi and Dr. Xuedong Yang (Division of Radiology, Guang’anmen Hospital, China Academy of Chinese Medical Sciences) for their help in imaging analysis. We also thank all the patients who donated their data for analysis and the medical staffs working in the front line.

## Author contributions

**Conceptualization:** Yang Gao, Guangxi Li.

**Data curation:** Yang Gao, Yuxiong Hu, Junteng Zhu, Huan Liu, Rongxian Qiu, Qunying Lin, Xiongzhi He, Shiming Cheng.

**Formal analysis:** Shiming Cheng.

**Funding acquisition:** Hai-Bin Lin.

**Investigation:** Huan Liu, Guangxi Li.

**Methodology:** Yang Gao, Huan Liu, Guangxi Li.

**Project administration:** Hai-Bin Lin, Shiming Cheng.

**Resources:** Yuxiong Hu, Junteng Zhu, Huan Liu, Rongxian Qiu, Qunying Lin, Xiongzhi He, Hai-Bin Lin.

**Supervision:** Hai-Bin Lin.

**Writing – original draft:** Yang Gao.

**Writing – review & editing:** Hai-Bin Lin, Guangxi Li.

## References

[1] WHO. Coronavirus disease (COVID-2019) situation reports. Available online: https://www.who.int/emergencies/diseases/novel-coronavirus-2019/situation-reports/ [accessed July 3, 2020]

[2] American College of Radiology (ACR). ACR recommendations for the use of chest radiography and computed tomography (CT) for suspected COVID-19 infection. Available online: https://www.acr.org/Advocacy-and-Economics/ACR-Position-Statements/Recommendations-for-Chest-Radiography-and-CT-for-Suspected-COVID19-Infection. Updated March 2020 [accessed May 24, 2020]

[3] National Health Commission of the People's Republic of China. The guidlines for the diagnosis and treatment of 2019-nCoV pneumonia (the 7th edition). Available at: http://www.nhc.gov.cn/yzygj/s7653p/202002/d4b895337e19445f8d728fcaf1e3e13a/files/ab6bec7f93e64e7f998d802991203cd6.pdf [accessed March 27, 2020]

[4] Rubin GD Ryerson CJ Haramati LB. The role of chest imaging in patient management during the COVID-19 pandemic: a multinational consensus statement from the Fleischner society. Chest 2020;158:106–16. Epub 2020 Apr 7. 32275978 10.1016/j.chest.2020.04.003 PMC7138384

[5] Wang L. C-reactive protein levels in the early stage of COVID-19. Med Mal Infect 2020;50:332–4. 32243911 10.1016/j.medmal.2020.03.007 PMC7146693

[6] Xie L Liu Y Xiao Y. Follow-up study on pulmonary function and lung radiographic changes in rehabilitating severe acute respiratory syndrome patients after discharge. Chest 2005;127:2119–24. 15947329 10.1378/chest.127.6.2119 PMC7094359

[7] Ajlan AM Ahyad RA Jamjoom LG. Middle East respiratory syndrome coronavirus (MERS-CoV) infection: chest CT findings. AJR Am J Roentgenol 2014;203:782–7. 24918624 10.2214/AJR.14.13021

[8] Zhou S Wang Y Zhu T. CT features of coronavirus disease 2019 (COVID-19) pneumonia in 62 patients in Wuhan, China. AJR Am J Roentgenol 2020;214:1287–94. Epub 2020 Mar 5. 32134681 10.2214/AJR.20.22975

[9] Bai HX Hsieh B Xiong Z. Performance of radiologists in differentiating COVID-19 from viral pneumonia on chest CT. Radiology 2020;200823 Epub ahead of print. 10.1148/radiol.2020200823 PMC7233414 32155105

[10] Ye Z Zhang Y Wang Y. Chest CT manifestations of new coronavirus disease 2019 (COVID-19): a pictorial review. Eur Radiol 2020;30:4381–9. Epub 2020 Mar 19. 32193638 10.1007/s00330-020-06801-0 PMC7088323

[11] Shi H Han X Jiang N. Radiological findings from 81 patients with COVID-19 pneumonia in Wuhan, China: a descriptive study. Lancet Infect Dis 2020;20:425–34. 32105637 10.1016/S1473-3099(20)30086-4 PMC7159053

[12] Miller R Englund K. Clinical presentation and course of COVID-19. Cleve Clin J Med 2020;87:384–8. 32371564 10.3949/ccjm.87a.ccc013

[13] Chen N Zhou M Dong X. Epidemiological and clinical characteristics of 99 cases of 2019 novel coronavirus pneumonia in Wuhan, China: a descriptive study. Lancet 2020;395:507–13. 32007143 10.1016/S0140-6736(20)30211-7 PMC7135076

[14] Bilgir O Bilgir F Calan M. Comparison of pre- and post-levothyroxine high-sensitivity c-reactive protein and fetuin-a levels in subclinical hypothyroidism. Clinics (Sao Paulo) 2015;70:97–101. 25789517 10.6061/clinics/2015(02)05 PMC4351305

[15] Chalmers S Khawaja A Wieruszewski PM. Diagnosis and treatment of acute pulmonary inflammation in critically ill patients: the role of inflammatory biomarkers. World J Crit Care Med 2019;8:59–71. 31559145 10.5492/wjccm.v8.i5.59 PMC6753396

[16] Warusevitane A Karunatilake D Sim J. Early diagnosis of pneumonia in severe stroke: clinical features and the diagnostic role of C-reactive protein. PLoS One 2016;11:e0150269. 26937636 10.1371/journal.pone.0150269 PMC4777448

[17] Yu J Zheng R Lin H. Changes of chest imaging of patients with influenza a H1N1 (Critical Care): a clinical research. Chin J Nosocomiol 2011;2:288–90.

[18] Zheng R Qiu H. Clinical study on the changes of chest imaging in severe acute respiratory syndrome. J Southeast Univ (Med Sci Edi) 2003;4:217–22+211.

[19] Chen Z Yang J Dai B. Forecast possible risk for COVID-19 epidemic dissemination under current control strategies in Japan. Int J Environ Res Public Health 2020;17:3872. 10.3390/ijerph17113872 PMC7312241 32486011

[20] Wang Y Dong C Hu Y. Temporal changes of CT findings in 90 patients with COVID-19 pneumonia: a longitudinal study. Radiology 2020;200843 Epub ahead of print. 10.1148/radiol.2020200843 PMC7233482 32191587

[21] Ojha V Mani A Pandey NN. CT in coronavirus disease 2019 (COVID-19): a systematic review of chest CT findings in 4410 adult patients. Eur Radiol 2020;1–0. Epub ahead of print. 10.1007/s00330-020-06975-7 PMC7261039 32474632

[22] Ai T Yang Z Hou H. Correlation of chest CT and RT-PCR testing in coronavirus disease 2019 (COVID-19) in China: a report of 1014 cases. Radiology 2020;200642 Epub ahead of print. 10.1148/radiol.2020200642 PMC7233399 32101510

[23] Fang Y Zhang H Xie J. Sensitivity of Chest CT for COVID-19: comparison to RT-PCR. Radiology 2020;200432 Epub ahead of print. 10.1148/radiol.2020200432 PMC7233365 32073353

[24] Koo HJ Lim S Choe J. Radiographic and CT features of viral pneumonia. Radiographics 2018;38:719–39. 29757717 10.1148/rg.2018170048

[25] Yang W Sirajuddin A Zhang X. The role of imaging in 2019 novel coronavirus pneumonia (COVID-19). Eur Radiol 2020;1–9 Epub ahead of print. 10.1007/s00330-020-06827-4 PMC7156903 32296940

[26] Zhu Y Gao ZH Liu YL. Clinical and CT imaging features of 2019 novel coronavirus disease (COVID-19). J Infect 2020;81:147–78. Epub 2020 Apr 8. 10.1016/j.jinf.2020.03.033 PMC7194958 32277968

[27] Raptis CA Hammer MM Short RG. Chest CT and coronavirus disease (COVID-19): a critical review of the literature to date. AJR Am J Roentgenol 2020;1–4. Epub ahead of print. 10.2214/AJR.20.23202 32298149

[28] Zhao W Zhong Z Xie X. CT scans of patients with 2019 novel coronavirus (COVID-19) pneumonia. Theranostics 2020;10:4606–13. 32292517 10.7150/thno.45016 PMC7150491

[29] Radford IR Murphy TK. Radiation response of mouse lymphoid and myeloid cell lines. Part III. Different signals can lead to apoptosis and may influence sensitivity to killing by DNA double-strand breakage. Int J Radiat Biol 1994;65:229–39. 7907120 10.1080/09553009414550261

[30] Trowell OA. The sensitivity of lymphocytes to ionising radiation. J Pathol Bacteriol 1952;64:687–704. 13000583 10.1002/path.1700640403

[31] Paces J Strizova Z Smrz D. COVID-19 and the immune system. Physiol Res 2020;69:379–88. 32469225 10.33549/physiolres.934492 PMC8648321

